# Clinical trials software: commercial system or build your own?

**DOI:** 10.1186/1745-6215-14-S1-P61

**Published:** 2013-11-29

**Authors:** Gladys McPherson, Mark Forrest, Sazid Mohammed

**Affiliations:** 1 University of Aberdeen, Aberdeen, Scotland, UK

## 

A question that continues to be asked by Clinical Trials Units is ‘Should we invest in commercial trials software or should we develop our own solutions?' The answer depends on the expertise available within the organisation, volume and diversity of work undertaken by the Unit and of course the resources available. The benefits of using electronic technologies are well known; they promote better access and analysis of data as well as providing study results electronically.

There are many commercial solutions available. These are often prohibitively expensive, require extensive training, management and support. They are not flexible or adaptable as there is usually no access to the source code so it is difficult to customise or extend the functionality.

Trials units conducting publicly funded trials who have access to an experienced programming team having knowledge and experience of good clinical practice and current legislation could consider developing their own generic software solution. The pros and cons of this approach will be explored in this presentation along with comparisons with some of the commercially available solutions.

The requirements for trial administration, data management, data cleaning, checking and extraction for analysis will be understood through experience and collaboration with trials teams. A well-documented and tested standard solution developed in-house can provide the benefits of commercial software coupled with the flexibility and quick response offered by a resident programming team. The professional expertise brought to a trials unit by a programming team with trials experience cannot be easily replaced by purchasing a software solution.

